# Warmth and competence in affective companion virtual humans: differential effects on behavioral intention via hedonic benefits

**DOI:** 10.3389/fpsyg.2026.1877802

**Published:** 2026-09-08

**Authors:** Ting Zhang

**Affiliations:** School of Humanities, Fujian University of Technology, Fuzhou, China

**Keywords:** affective companion virtual humans, behavioral intention, hedonic benefits, perceived competence, perceived warmth, privacy cynicism

## Abstract

Generation Z increasingly engages with affective companion virtual humans not as tools but as partners. However, it remains unclear how social perceptions—specifically warmth and competence—privacy-related attitudes jointly influence their behavioral intention, and whether hedonic benefits mediate these effects. Drawing on the hedonic motivation system acceptance model and the stereotype content model, this study proposes an integrated model linking perceived warmth, perceived competence, perceived privacy risk and privacy cynicism to behavioral intention toward affective companion virtual humans, with hedonic benefits as a mediator. Using partial least squares structural equation modeling (PLS-SEM) on 348 valid responses from a Generation Z sample, the results show that: (1) perceived warmth, perceived competence and privacy cynicism are directly and positively associated with behavioral intention; (2) hedonic benefits partially mediate the effect of perceived warmth on behavioral intention (VAF = 36%), but play only a negligible mediating role for perceived competence (VAF = 15%); and (3) perceived privacy risk has no significant direct or indirect effect. These findings clarify the differential pathways of warmth and competence in affective companion virtual humans acceptance: warmth is associated with both direct and hedonic routes, whereas competence is predominantly associated with intention through direct instrumental pathways. The results also indicate that privacy cynicism can co-exist with continued use intention without diminishing hedonic experience. Practically, the findings suggest that enhancing emotional warmth and functional competence, while acknowledging users' privacy fatigue, is more effective than solely focusing on reducing privacy risk perceptions.

## 1 Introduction

Recent years have witnessed a growing body of research on AI systems designed for emotional interaction, giving rise to a range of related but fragmented concepts—such as “affective companion agents” (Gong and Wang, 2025), “emotional companions” (Xie et al., 2025), and “chatbot companionship” (Bittner et al., 2025). These efforts collectively reflect an emerging scholarly consensus that AI is increasingly expected to play a sustained socio-emotional role in users' lives (Sun and Wu, 2025). Building on and synthesizing these developments, we propose a more integrated term—Affective companion virtual humans (ACVH), —to refer specifically to conversational AI systems engineered for continuous, affectively charged companionship. In this study, we define ACVH as AI systems designed to provide ongoing emotional support through empathetic, personalized, and long-term interactions. Unlike task-oriented chatbots, ACVH are designed to simulate warmth, empathy and social presence, appealing to users' needs for emotional connection and self-disclosure (Deng and Yan, 2025; Merrill et al., 2025). Unlike avatars (user-controlled self-representations), virtual agents (task-oriented characters), and virtual influencers (commercial branding characters), ACVH are distinguished by their primary goal of fulfilling users' socio-emotional needs through sustained, affectively charged interaction. For Generation Z (born 1997–2012)—the first generation to grow up with the international technologies as an integral part of daily life—ACVH are frequently perceived by Generation Z as “partners” rather than mere “tools” (Pataranutaporn et al., 2025).

Prior research has examined how social perceptions—especially warmth and competence, influence users' trust in and acceptance of AI agents (Harris-Watson et al., 2023; Carpinella et al., 2017). Other work has explored how anthropomorphic cues, such as empathy and social presence, shape user engagement and satisfaction (Deng and Yan, 2025; Merrill et al., 2025). Almokdad et al. (2026) further demonstrate that socially cued AI interfaces can enhance social presence, although they note that such presence does not directly reduce perceptions of service dehumanization—suggesting that the effects of social design features on user responses may depend on the service context and task type. Scholars have also begun to investigate privacy concerns associated with disclosing personal information to conversational agents (McLean and Osei-Frimpong, 2019; May and Denecke, 2022), as well as the paradoxical phenomenon of privacy cynicism—a resigned belief that privacy protection is futile, which has been shown to coexist with continued use in social media contexts (Khan et al., 2023; Hoffmann et al., 2016). More recently, studies have highlighted the potential benefits of AI companionship for emotional wellbeing (Pataranutaporn et al., 2025; Liu et al., 2025), while also noting that many ACVH products remain limited by superficial interactivity and generic emotional responsiveness (Smith et al., 2025; Sung and Ferdman, 2025).

Recent meta-analyses and systematic reviews have consolidated this fragmented body of evidence. A large-scale meta-analysis of text-based chatbots found that human-like social cues exert a small-to-medium positive effect on users' social responses, although the effect is moderated by user characteristics, agent types, and interaction contexts (Klein, 2025). Similarly, a meta-analysis of conversational bot acceptance identified perceived usefulness, ease of use, trust, and satisfaction as consistent drivers of acceptance, while anthropomorphic features enhanced enjoyment but did not necessarily foster trust (Fares and Lee, 2026). These quantitative syntheses suggest that the effects of social cues are more nuanced than previously assumed, underscoring the need to examine how specific social perceptions—particularly warmth and competence—operate through distinct mechanisms in affective companionship contexts.

Regarding AI companionship specifically, recent empirical work has revealed both the promises and limits of these technologies. De Freitas et al. (2025) demonstrated that while people perceive AI companions as more available and non-judgmental than humans, they resist viewing them as capable of mutual caring—an essential value of authentic relationships. This belief in “relationship inauthenticity” persisted even after actual interaction with AI companions. Meanwhile, a mixed-methods study on AI companionship attitudes found that 55.6% of participants expressed hesitancy toward emotionally significant AI relationships, citing lack of physicality, lack of humanity, and incapacity to form emotional connection as key barriers, while also acknowledging that non-judgmental support and emotional availability were the primary motivations for engagement (Vowels et al., 2025). These findings collectively suggest that while AI companions offer potential benefits—including non-judgmental support and emotional availability—significant psychological barriers remain, which may be shaped by users' social perceptions of warmth and competence.

Classic technology acceptance models have long identified perceived usefulness, ease of use and hedonic motivation as core determinants of technology adoption (Davis, 1989; Venkatesh et al., 2012). In parallel, recent HCI research has shown that social perception dimensions—particularly warmth and competence—significantly influence human trust and acceptance of AI agents (Harris-Watson et al., 2023; Carpinella et al., 2017). Studies in the human-computer interaction further suggest that user-friendliness and playfulness-representing utilitarian and hedonic values, respectively—predict continuance intention toward generative AI chatbots (Kim and Baek, 2025), underscoring the importance of hedonic experiences in sustaining user engagement with conversational AI.

Despite these advances, several important gaps remain. First, existing studies predominantly examine warmth and competence in isolation or as parallel predictors, without clarifying how these perceptions operate through hedonic mechanisms in an affective companionship context. Second, although privacy risk is commonly assumed to deter acceptance, the role of privacy cynicism as a potential *positive* driver of continued use—through a “trade-off” logic—has not been systematically tested in ACVH settings. Third, the specific pathways through which warmth and competence translate into behavioral intention via hedonic benefits remain underexplored. Emerging studies have begun to explore the role of chatbot empathy in shaping user outcomes (Jiang et al., 2025; Smith et al., 2025; Sung and Ferdman, 2025), yet the mechanisms linking warmth and competence to behavioral intention via hedonic benefits remain insufficiently specified in the ACVH literature. Consequently, how warmth, competence, privacy risk, and cynicism jointly shape users' behavioral intention toward ACVH remains unclear, which directly motivates the present investigation.

To address these gaps, this study proposes an integrated model that incorporating perceived warmth, perceived competence, perceived privacy risk, and privacy cynicism as antecedents, behavioral intention as the outcome, and hedonic benefits as a mediator. Focusing specifically on Generation Z, we pose the following research questions:

*RQ1: Do perceived warmth, perceived competence, and privacy cynicism directly influence behavioral intention toward ACVH?*
*RQ2: Do hedonic benefits mediate* the relationships between these antecedents and *behavioral intention?*

### 1.1 Theoretical framework

Our theoretical framework draws on three complementary perspectives. Consistent with the hedonic motivation system acceptance model (HMSAM; Venkatesh et al., 2012), we conceptualize hedonic benefits as a proximal driver of behavioral intention. Drawing on the stereotype content model (SCM; Fiske et al., 2007), we argue that both warmth and competence matter, yet they are likely to operate through distinct mechanisms: warmth may primarily enhance hedonic enjoyment, whereas competence may also signal instrumental reliability and usefulness. Following the privacy cynicism literature (Khan et al., 2023; Hoffmann et al., 2016), we propose a counterintuitive mechanism: users who cynically accept privacy risks may nevertheless sustain usage intention, provided that hedonic benefits are sufficiently salient.

The integration of these three perspectives follows a clear theoretical logic. First, HMSAM provides the baseline mechanism explains how hedonic benefits drive behavioral intention. Second, the stereotype content model explains how users perceive ACVH's social attributes—specifically, warmth and competence. Third, privacy cynicism theory accounts for how users trade off privacy concerns against service benefits. These perspectives are conceptually complementary: SCM identifies which social perceptions matter when users treat ACVH as social actors—an assumption consistent with the media equation theory (Reeves and Nass, 1996), which posits that individuals respond to media as if they were human; HMSAM explains how these perceptions translate into intention through hedonic benefits as an intrinsic motivational pathway, and privacy cynicism theory accounts for why privacy-related attitudes can coexist with continued use, framing cynicism as a coping response rather than a deterrent. Together, these perspectives constitute an integrated pathway in which social perceptions shape emotional experience, which in turn influences behavioral intention—a sequence that we contend is particularly relevant for affective technologies wherein emotional engagement is central.

### 1.2 Research contributions

This study offers three contributions. First, it empirically disentangles the direct and indirect effects of warmth and competence on ACVH acceptance, demonstrating that warmth operates through both direct and hedonic pathway, whereas competence exerts a predominantly direct effect with only negligible mediation. This context-sensitive differentiation extends prior research on human-AI teaming (Harris-Watson et al., 2023) by specifying the mechanisms rather than merely the presence of these effects. Second, it clarifies the mediating function of hedonic benefits for social perception variables, showing that hedonic benefits partially mediate the warmth-intention relationship while playing a negligible role for competence, thereby reinforcing HMSAM in an under explored ACVH context. Third, it provides initial evidence that privacy cynicism may function as a positive rather than strictly negative predictor of behavioral intention in emotional companion AI, extending the privacy paradox research beyond social media (Khan et al., 2023; Hoffmann et al., 2016) to human-AI emotional interaction-a domain where privacy concerns involve deeper emotional self-disclosure rather than mere data sharing.

## 2 Conceptual development and research hypotheses

**Figure 1:**
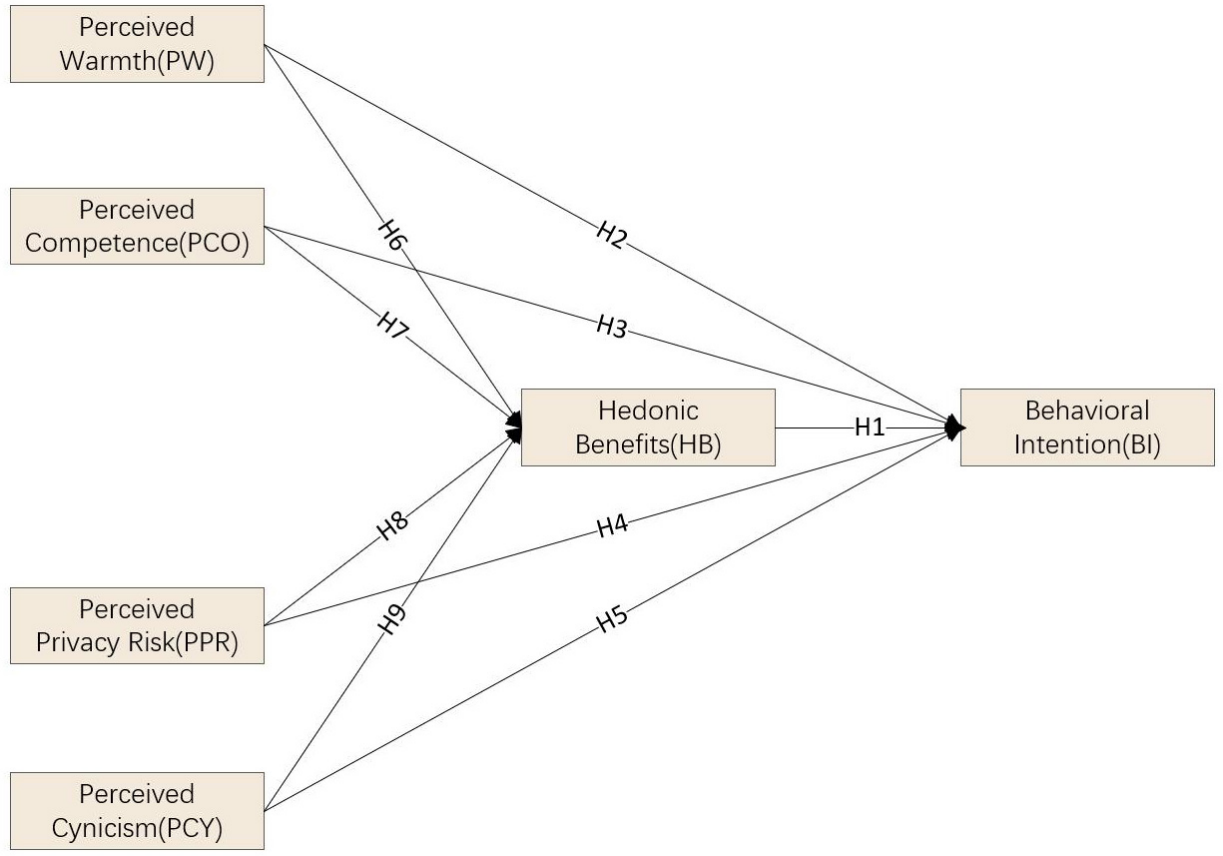
Research model and hypotheses.

Behavioral intention denotes an individual's willingness or inclination to use a specific technology, serving as a fundamental indicator for assessing technology acceptance (Marikyan and Papagiannidis, 2021). As a pivotal variable in technology acceptance models, behavioral intention not only reflects users' intrinsic motivation for usage but also demonstrates a strong predictive validity with actual technology adoption behavior, establishing it as a central focus in technology acceptance research. To systematically investigate the influencing mechanisms underlying Generation Z's behavioral intention toward ACVH, this study develops a theoretical framework that integrates social perception factors and risk attitude variables, grounded in the Hedonic Motivation System Acceptance Model (HMSAM). HMSAM posits that hedonic benefits, as a form of intrinsic motivation, play a critical role in driving technology behavioral intention (Venkatesh et al., 2012); however, the specific pathways through which these benefits operate in the context of ACVH require further exploration. This study hypothesizes that Generation Z's perceptions of warmth, competence, and privacy risk, along with their attitude of privacy cynicism, may not only directly influence behavioral intention toward ACVH but also exert indirect effects via hedonic benefits. Consequently, this study establishes an integrated model encompassing social perception (perceived warmth, perceived competence, perceived privacy risk), attitude (privacy cynicism), a mediating variable (hedonic benefits), and an outcome variable (behavioral intention) (see Figure 1 for the research framework).

### 2.1 Behavioral Intention

The Technology Acceptance Model (TAM) initially identified behavioral intention as a mediating factor influencing actual usage behavior, serving to mediate the effects of antecedent factors on usage behavior (Davis, 1989). Over time, behavioral intention has evolved into a primary dependent variable in technology acceptance research, directly reflecting users' willingness to adopt technology. In the realm of ACVH, the behavioral intention of Generation Z is pivotal not only for enhancing human-AI interaction and informing product design but also because its current low level presents a substantial barrier to the widespread adoption. Therefore, identifying the key factors that influence this intention is of considerable theoretical and practical importance.

Previous research has identified three primary dimensions influencing technology acceptance: demographic characteristics, psychological traits, and behavioral aspects. In the realm of AI adoption, attitudes, trust, social cognition, and hedonic benefits have emerged as significant predictors, prompting an increasing scholarly focus on the complex interrelations among these factors. Recent studies indicate that perceived warmth and competence are critical in teams' acceptance of AI partners (Harris-Watson et al., 2023), while HMSAM highlights the mediating role of hedonic motivation in technology acceptance (Venkatesh et al., 2012). Building on these foundations, this study posits that various psychological factors affect the behavioral intention, with hedonic motivation serving as a mediator.

### 2.2 Hedonic benefits

Hedonic benefits refer to the emotional value, including pleasure and enjoyment, that users derive from engaging with technology. In addition to fulfilling users' self-actualization needs (Van der Heijden, 2004), these benefits can enhance active user engagement by providing innovative and captivating interactive experiences. ACVH, as a technological embodiment that merges entertainment with companionship, inherently addresses the hedonic needs of Generation Z through its diverse interactive scenarios and anthropomorphic interaction modes. Extended versions of technology acceptance models, such as TAM2 and UTAUT, have empirically validated the crucial role of hedonic motivation in technology adoption. Moreover, HMSAM further elucidates its significance as a fundamental intrinsic motivation (Venkatesh et al., 2012). Through conversational interactions with ACVH, users not only experience unexpected delight from novel technology but also attain emotional satisfaction through sustained engagement. These positive experiences reinforce the relationship between ACVH and hedonic value, thereby enhancing users' behavioral intention. Consequently, this study proposes the following hypothesis:

***H1: The hedonic benefits of ACVH significantly influence behavioral*** ***intention*.**

### 2.3 Perceived warmth

Warmth is a fundamental component of social cognition, characterized by traits such as friendliness, empathy, and trustworthiness, which often outweigh competence in fostering interpersonal collaboration (Casciaro and Lobo, 2005). This perspective diverges from the traditional emphasis on functional competence in technology acceptance research (Cuddy et al., 2011). Studies indicate that perceived warmth is vital in building trust in AI, as it facilitates the development of positive relational connections, including trust and a sense of belonging (Kulms and Kopp, 2018; Christoforakos et al., 2021). The warmth of ACVH as social entities significantly influences users' emotional responses and their willingness to engage in interactions. In contexts requiring emotional companionship, fostering warm interactive experiences is crucial for establishing favorable human-machine relationships and promoting emotional wellbeing (Pelau et al., 2021). Although direct empirical investigations into the impact of perceived warmth on behavioral intention in ACVH are limited, research on interpersonal interactions strongly supports the importance of perceived warmth in cultivating trust and positive attitudes. Based on this theoretical framework, ACVH perceived as warmer are more likely to resonate emotionally with users, thereby enhancing their behavioral intention. Consequently, this study proposes the following hypothesis:

***H2: Perceived warmth of ACVH has a significant positive effect on behavioral*** ***intention*.**

### 2.4 Perceived competence

Perceived competence refers to users' assessments of ACVH's ability to address problems and provide effective services, forming the essential basis for technological adoption (Kulms and Kopp, 2018; Christoforakos et al., 2021). From TAM to its subsequent extensions, perceived usefulness—essentially an elaboration of the competence dimension—has consistently been a critical predictor of technology acceptance. In the context of human-AI interactions, ACVH maintains an instrumental role, and its ability to effectively meet users' emotional needs while delivering accurate responses significantly impacts users' attitudes and willingness to continue use. Empirical research has demonstrated that perceived competence enhances human trust in AI agents (Gillath et al., 2021). When users first interact with ACVH, recognizing its strong problem-solving abilities and seamless functionality is likely to result in positive evaluations and an increased willingness to continue using the technology. Consequently, this study proposes the following hypothesis:

***H3: Perceived competence of ACVH has a significant positive effect on*** ***behavioral intention*.**

### 2.5 Perceived privacy risk

The widespread proliferation of network technologies has rendered privacy risk a central ethical concern in AI applications. When interacting with ACVH, users must disclose personal privacy information, either actively or passively, which is frequently utilized for model training. Despite service providers' claims regarding the implementation of privacy protection measures, perceived privacy risk remains a critical determinant of users' attitudes toward ACVH. Empirical evidence indicates that perceived privacy risk undermines the actual benefits users derive from AI services, consequently diminishing their willingness to engage with these technologies (Harris-Watson et al., 2023). Users' apprehensions about the security of privacy information transmission and the potential for unauthorized use directly dampen their enthusiasm for interacting with ACVH, potentially leading to the cessation of usage behavior. Therefore, this study proposes the following hypothesis:

***H4: Perceived privacy risk of ACVH has a significant negative effect on*** ***behavioral intention*.**

### 2.6 Privacy cynicism

Privacy cynicism is defined as “an attitude characterized by uncertainty, powerlessness, and distrust toward online service providers' personal data processing practices, with individuals subjectively perceiving privacy protection behaviors as ineffective” (Khan et al., 2023; Hoffmann et al., 2016). This attitude reflects a defensive cognitive strategy that users employ to navigate online privacy risks. It arises from users' beliefs that privacy breaches are unavoidable or from the trade-off of “exchanging personal information for services”, ultimately resulting in a compromise between perceived privacy risks and the benefits obtained from the service. In the context of ACVH usage, users acknowledge the presence of privacy risks but do not outright reject the technology; rather, they adopt a cautious stance. This behavior is consistent with the compromise logic inherent in privacy cynicism. The emotional companionship value provided by ACVH encourages some users to prioritize service benefits over privacy concerns, thus maintaining their behavioral intention. Consequently, this study proposes the following hypothesis:

***H5: Privacy cynicism has a significant positive effect on behavioral intention*** ***toward ACVH*.**

### 2.7 Mediation effect of hedonic benefits

#### 2.7.1 Perceived warmth and hedonic benefits

ACVH characterized by high perceived warmth offers users emotional experiences marked by feelings of respect and understanding through friendly and empathetic interactions, thereby addressing their needs for belonging and care. This relaxed and stress-free mode of interaction enables users to maintain positive emotions, reduce anxiety, and build positive emotional experiences, which in turn enhances their perception of hedonic benefits. Consequently, this study posits the following hypothesis:

***H6: Perceived warmth of ACVH significantly positively influences hedonic*** ***benefits*.**

#### 2.7.2 Perceived competence and hedonic benefits

ACVH's high perceived competence is evident in its capacity to swiftly comprehend user needs, deliver precise responses, and offer efficient services. This seamless interaction experience can elevate users' positive emotions and enhance their emotional communication contexts. Robust functional competence provides users with convenient emotional support, broadens the opportunities for emotional companionship, and thereby continually enhances hedonic benefits. Consequently, this study posits the following hypothesis:

***H7: The perceived competence of ACVH significantly positively influences*** ***hedonic benefits*.**

#### 2.7.3 Perceived privacy risk and hedonic benefits

Perceived privacy risk engenders a trust deficit among users regarding ACVH, prompting them to adopt a cautious approach during interactions. Users experience anxiety due to concerns about privacy breaches and unauthorized usage, as well as excessive self-censorship that restricts genuine emotional expression. This anxiety diminishes the enjoyment of interactions, reduces the positive emotional experiences associated with usage, and ultimately undermines the perception of hedonic benefits. Consequently, this study posits the following hypothesis:

***H8: Perceived privacy risk of ACVH has a significant negative effect on*** ***hedonic benefits*.**

#### 2.7.4 Privacy cynicism and hedonic benefits

The feelings of powerlessness and distrust linked to privacy cynicism impede users from fully engaging in interactions with ACVH and from fostering positive human-machine relationships. This detrimental mindset generates anxiety, diminishes users' willingness to express themselves freely, and obstructs the attainment of positive emotional experiences during interactions, ultimately undermining hedonic benefits. Consequently, this study posits the following hypothesis:

***H9: Privacy cynicism has a significant negative effect on the hedonic benefits*** ***of ACVH*.**

## 3 Method

### 3.1 Participants and procedure

To test the proposed model, an online survey was administered to Generation Z participants (born 1997–2012) through Wenjuanxing (www.wjx.cn), a leading online survey platform in China. To ensure that all participants shared a consistent understanding of the target construct, the survey opened with a brief description of ACVH, explicitly defining them as conversational AI systems designed for continuous emotional companionship—distinguishing them from generic task-oriented chatbots (e.g., customer service bots or informational assistants)—and providing concrete examples of typical applications (e.g., Replika, Xiaoice, emotional support chatbots). Participation was voluntary and anonymous, and no incentives were provided. Ethical guidelines for social science research were followed throughout.

A total of 414 responses were collected over 1 week. After excluding 66 invalid responses (e.g., completion time < 2 min, straight-lining, identical answers across all items), 348 valid responses remained, yielding an effective response rate of 84.1%. To ensure adequate statistical power, we conducted an a priori power analysis using G^*^Power 3.1 (Faul et al., 2009). For linear multiple regression with a maximum of five predictors—the largest number of paths pointing to any endogenous construct in our model—a medium effect size (*f* ^2^ = 0.15), α = 0.05, and desired power (1–β) = 0.80, the minimum required sample size was 92. Our sample of 348 substantially exceeds this requirement, providing sufficient power to detect medium-to-small effects. Additionally, following Hair et al. (2019) 10-times rule for PLS-SEM (10 times the maximum number of paths pointing to any construct, which is five), the minimum requirement is 50. Our sample of 348 is well above both criteria.

Table 1 summarizes the demographic characteristics. The sample included 64.7% female and 35.3% male participants. The majority (88.5%) were aged 18–25 years, and 11.5% aged 26–30. In terms of education, 75.9% held or were pursuing a bachelor's degree, and 15.2% held or were pursuing a master's degree.

**Table 1:** Demographic characteristic variables of respondents.

Demographic characteristic variables		*N*	%
Gender	Female	225	64.7
	Male	123	35.3
Age/year	18–25	308	88.5
	26–30	40	11.5
Education	High school and below	5	1.4
	College degree	24	6.9
	Bachelor's degree/Master's degree in progress	264	75.9
	Master's degree/Doctor's degree in progress	53	15.2
	Doctor's degree and above	2	0.6

N, Frequency; %, Percentage.

According to the regulations of the Science and Technology Ethics Committee of Fujian University of Technology, formal ethics approval was not required for this anonymous survey-based study as it involved no more than minimal risk to participants and did not collect identifiable personal information.

All procedures were conducted in accordance with the Declaration of Helsinki. Informed consent was obtained from all participants by informing them that completion and submission of the questionnaire implied consent. No personally identifiable information was collected, and no individual participants can be identified from this manuscript.

### 3.2 Measures

All constructs were measured using well-validated scales from previous research, adapted slightly to the ACVH context. A 7-point Likert scale (1 = strongly disagree, 7 = strongly agree) was used for all items. The full list of items is presented in Table 2.

**Table 2:** List of constructs and their items.

Construct	Scale items	Source
Hedonic benefits (HB)	HB1: Chatting with the ACVH is enjoyable. HB2: Chatting with the ACVH is fun. HB3: Chatting with the ACVH is pleasant. HB4: I like the feeling of interacting with the ACVH. HB5: Interacting with the ACVH is exciting.	Qiu and Benbasat, 2009; McLean and Osei-Frimpong, 2019
Perceived warmth (PW)	PW1: ACVH is enthusiastic. PW3: ACVH is friendly. PW4: Reply of ACVH is compassionate. PW5: Reply of ACVH is amicable.	Chattaraman et al., 2019; Shi and Jiang, 2023
Perceived competence (PCO)	PCO1: ACVH is competent. PCO2: ACVH is efficient. PCO3: ACVH is skillful. PCO4: ACVH is confident to complete instructions. PCO5: ACVH is sure of completing instructions.	Shi and Jiang, 2023
Perceived privacy risk (PPR)	PPR1: I am concerned that ACVH may share my personal information with third parties without my consent. PPR2: I am concerned to trade through ACVH. PPR3: I am concerned that the personal information I disclose to ACVH may be stolen. PPR4: I am concerned that the ACVH collects too much information about me. PPR5: I suspect that ACVH will collect information about me.	McLean and Osei-Frimpong, 2019
Privacy cynicism (PCY)	PCY1: I feel that no matter how hard I try, I cannot fully protect my online privacy. PCY2: With the development of technology, my online privacy will become increasingly accessible to others. PCY3: I feel that the invasion of personal privacy by the online world is inevitable. PCY4: I have become more cynical about whether my efforts in protecting online privacy are in any way effective. PCY5: My efforts to protect online privacy may become increasingly ineffective.	Khan et al., 2023
Behavioral intention (BI)	BI1: I intend to use ACVH if I can access. BI2: I predict that I will use ACVH if I can access. BI3: I will plan to use ACVH if I can access. BI4: I will use ACVH in the near future if I can access. BI5: I plan to use ACVH frequently in the near future if I can access.	Qiu and Benbasat, 2009; Gkartzonikas et al., 2023

***Behavioral intention (BI)*** was measured with five items adapted from Qiu and Benbasat (2009) and Gkartzonikas et al. (2023), capturing users' willingness and plans to use ACVH (e.g., “I intend to use ACVH if I can access it”).

***Hedonic benefits (HB)*** were assessed using five items from Qiu and Benbasat (2009) and McLean and Osei-Frimpong (2019), capturing enjoyment and pleasure (e.g., “Chatting with the ACVH is enjoyable”).

***Perceived warmth (PW)*** was measured with four items (after dropping one item with low factor loading in pilot testing) adapted from Chattaraman et al. (2019) and Shi and Jiang (2023), reflecting friendliness, empathy, and trustworthiness (e.g., “ACVH is friendly”).

***Perceived competence (PCO)*** used five items from Shi and Jiang (2023), reflecting the ACVH‘s ability to solve problems and deliver effective services (e.g., “ACVH is competent”).

***Perceived privacy risk (PPR)*** was measured with five items from McLean and Osei-Frimpong (2019), capturing concerns about data sharing and unauthorized use (e.g., “I am concerned that ACVH may share my personal information with third parties”).

***Privacy cynicism (PCY)*** was measured with five items from Khan et al. (2023), reflecting feelings of powerlessness and distrust regarding online privacy (e.g., “I feel that no matter how hard I try, I cannot fully protect my online privacy”).

A pilot study with 103 Generation Z respondents was conducted prior to the main survey to assess clarity and initial reliability. Minor wording adjustments were made based on feedback. Exploratory factor analysis (EFA) led to the removal of one perceived warmth item (PW2, factor loading < 0.60). The final measurement model demonstrated acceptable psychometric properties in the pilot.

### 3.3 Data analysis strategy

Partial least squares structural equation modeling (PLS-SEM) was employed using SmartPLS 4.0. PLS-SEM was selected over covariance-based SEM (CB-SEM) for two reasons. First, our research objective is prediction-oriented and theory development (rather than confirmation of a well-established theory), which aligns with the recommended use of PLS-SEM (Hair et al., 2019). Second, the data did not fully satisfy the multivariate normality assumption required for CB-SEM (Mardia's multivariate skewness and kurtosis tests were significant, *p* < 0.05), making PLS-SEM a more appropriate choice. A two-step approach was followed: (1) assessment of the measurement model (reliability, convergent validity, discriminant validity), and (2) assessment of the structural model (path coefficients, *R*^2^, effect sizes). Bootstrapping with 5,000 resamples was used to test the significance of path coefficients and mediation effects, employing bias-corrected and accelerated (BCa) bootstrap confidence intervals to assess the significance of indirect effects.

## 4 Results

### 4.1 Measurement model assessment

#### 4.1.1 Reliability and convergent validity

Table 3 summarizes the reliability and convergent validity of the measurement model. All constructs exhibited satisfactory internal consistency: Cronbach's α values ranged from 0.846 to 0.943, and composite reliability (CR) values ranged from 0.852 to 0.943, both exceeding the recommended threshold of 0.70. Average variance extracted (AVE) values ranged from 0.591 to 0.767, all above 0.50, supporting convergent validity. Item factor loadings all exceeded 0.69 (see Table 2), meeting the recommended criterion.

**Table 3:** Scales for reliability and validity of measurement model.

Construct	Item	M	SD	FL	α	CR	AVE
Hedonic benefits (HB)	HB1	4.94	1.263	0.857	0.918	0.919	0.696
	HB2	5.01	1.328	0.830			
	HB3	4.68	1.391	0.850			
	HB4	4.80	1.458	0.844			
	HB5	4.66	1.526	0.787			
Perceived warmth (PW)	PW1	5.14	1.298	0.747	0.846	0.852	0.591
	PW3	5.33	1.260	0.813			
	PW4	4.51	1.479	0.699			
	PW5	5.21	1.243	0.810			
Perceived competence (PCO)	PCO1	5.21	1.302	0.699	0.888	0.890	0.618
	PCO2	5.29	1.293	0.792			
	PCO3	4.81	1.362	0.798			
	PCO4	4.94	1.284	0.801			
	PCO5	4.96	1.276	0.835			
Perceived privacy risk (PPR)	PPR1	5.26	1.427	0.740	0.910	0.911	0.674
	PPR2	4.96	1.546	0.720			
	PPR3	5.19	1.458	0.855			
	PPR4	5.30	1.471	0.903			
	PPR5	5.35	1.480	0.871			
Privacy cynicism (PCY)	PCY1	5.52	1.411	0.762	0.886	0.892	0.609
	PCY2	5.52	1.421	0.758			
	PCY3	5.47	1.353	0.731			
	PCY4	5.36	1.348	0.824			
	PCY5	5.32	1.336	0.822			
Behavioral intention (BI)	BI1	5.31	1.177	0.861	0.943	0.943	0.767
BI2	5.35	1.208	0.888
BI3	5.30	1.181	0.877
BI4	5.40	1.196	0.868
BI5	5.32	1.210	0.884

M, mean; SD, standard deviation; FL, factor loading; α, Cronbach's alpha; CR, composite reliability; AVE, average variance extracted. Item loadings for PW4 and PCO1 are marginally below 0.70 (0.699), but are retained as the differences are negligible and all construct-level AVE and CR values exceed recommended thresholds.

#### 4.1.2 Discriminant validity

Discriminant validity was assessed using the Fornell-Larcker criterion (Table 4) and the HTMT ratio of correlations (Table 4a). For the Fornell-Larcker criterion, the square root of each construct's AVE (diagonal elements) exceeded its correlations with other constructs (off-diagonal elements). All HTMT values were below 0.85, the conservative threshold recommended by Henseler et al. (2015). These results confirm adequate discriminant validity.

**Table 4:** Discriminant validity and correlation matrix (Fornell-Larcker criterion).

Construct	HB	PW	PCO	PPR	PCY	BI
HB	**0.834**					
PW	0.655	**0.769**				
PCO	0.577	0.570	**0.786**			
PPR	0.132	0.112	0.160	**0.821**		
PCY	−0.005	0.045	0.064	0.478	**0.780**	
BI	0.464	0.463	0.519	0.187	0.223	**0.876**

Bold-faced diagonal elements are the square roots of AVEs. The off-diagonal elements are the correlations between constructs. HB, Hedonic benefits; BI, Behavioral intention; PW, Perceived warmth; PCO, Perceived competence; PPR, Perceived privacy risk; PCY, Privacy cynicism.

**Table 4a:** Heterotrait-Monotrait (HTMT) ratio of correlations.

Construct pair	HTMT value	95% CI	Status
HB ↔ BI	0.492	[0.388, 0.592]	< 0.85
PW ↔ HB	0.734	[0.654, 0.805]	< 0.85
PW ↔ BI	0.515	[0.404, 0.624]	< 0.85
PCO ↔ HB	0.628	[0.538, 0.714]	< 0.85
PCO ↔ BI	0.562	[0.468, 0.654]	< 0.85
PCO ↔ PW	0.653	[0.562, 0.737]	< 0.85
PPR ↔ HB	0.150	[0.042, 0.258]	< 0.85
PPR ↔ BI	0.201	[0.088, 0.314]	< 0.85
PPR ↔ PW	0.136	[0.028, 0.248]	< 0.85
PPR ↔ PCO	0.180	[0.066, 0.292]	< 0.85
PCY ↔ HB	0.072	[0.000, 0.156]	< 0.85
PCY ↔ BI	0.236	[0.118, 0.356]	< 0.85
PCY ↔ PW	0.090	[0.000, 0.188]	< 0.85
PCY ↔ PCO	0.092	[0.000, 0.186]	< 0.85
PCY ↔ PPR	0.529	[0.428, 0.626]	< 0.85

All HTMT values are below the conservative threshold of 0.85 (Henseler et al., 2015). The HTMT for PPR ↔ PCY was 0.529 (95% CI [0.428, 0.626]), confirming adequate discriminant validity between perceived privacy risk and privacy cynicism.

#### 4.1.3 Common method bias

Common method bias was assessed using Harman's single-factor test. An unrotated exploratory factor analysis extracted seven factors with eigenvalues > 1.0. The first factor explained 33.42% of the total variance, well below the 50% threshold, In addition, we performed a full collinearity test using inner VIF values (Kock, 2015). All inner VIFs were below the conservative threshold of 3.3 (range: 1.07–1.92), further indicating that common method bias does not substantially affect the results.

#### 4.1.4 Multicollinearity

Multicollinearity was assessed using variance inflation factors (VIFs). All VIF values were below 2.0 (range: 1.07–1.92), indicating no problematic collinearity.

### 4.2 Structural model assessment

Model Fit. Table 5 reports the model fit indices. The SRMR = 0.040 (< 0.08), TLI = 0.937 (> 0.90), and CFI = 0.944 (> 0.90). This value indicate good model fit.

**Table 5:** Model fit summary.

Fit indices	SRMR
Inspection value	0.040
Reference value	< 0.08

#### 4.2.1 Hypothesis testing

Table 6 summarizes the hypothesis testing outcomes. Key findings are as follows:

**Table 6:** The results of hypothesis testing.

Dependent variable	Hypothesis	Path	S.E.	*R* ^2^	β	*P* value	Hypothesis supported
BI	H1	HB → BI	0.075	0.363	0.181^*^	*p* < 0.05(^**^)	YES
BI	H2	PW → BI	0.091		0.157^*^	*p* < 0.05(^**^)	YES
BI	H3	PCO → BI	0.081		0.312^***^	*p* < 0.001(^**^)	YES
BI	H4	PPR → BI	0.056		−0.002 n/s	n.s.(*p* > 0.05)	NO
BI	H5	PCY → BI	0.056		0.196^***^	*p* < 0.001(^**^)	YES
HB	H6	PW → HB	0.079	0.496	0.483^***^	*p* < 0.001(^**^)	YES
HB	H7	PCO → HB	0.076		0.296^***^	*p* < 0.001(^**^)	YES
HB	H8	PPR → HB	0.054		0.067 n/s	n.s.(*p* > 0.05)	NO
HB	H9	PCY → HB	0.054		−0.078 n/s	n.s.(*p* > 0.05)	NO

^*^p < 0.05; ^**^p < 0.01; ^***^p < 0.001; n/s, not significant. HB, Hedonic benefits; BI, Behavioral intention; PW, Perceived warmth; PCO, Perceived competence; PPR, Perceived privacy risk; PCY, Privacy cynicism.

Direct effects on behavioral intention:

Hedonic benefits (H1) was positively associated with behavioral intention (β = 0.181, *p* < 0.05).

Perceived warmth (H2) was positively associated with behavioral intention (β = 0.157, *p* < 0.05).

Perceived competence (H3) was positively associated with behavioral intention (β = 0.312, *p* < 0.001).

Privacy cynicism (H5) significantly and positively associated with behavioral intention (β = 0.196, *p* < 0.001).

Perceived privacy risk (H4) showed no significant effect on behavioral intention (β = 0.002, n.s.).

Direct effects on hedonic benefits:

Perceived warmth (H6) was positively associated with hedonic benefits (β = 0.483, *p* < 0.001).

Perceived competence (H7) was positively associated with hedonic benefits (β = 0.296, *p* < 0.001).

Perceived privacy risk (H8) showed no significant effect on hedonic benefits (β = 0.067, n.s.).

**Figure 2:**
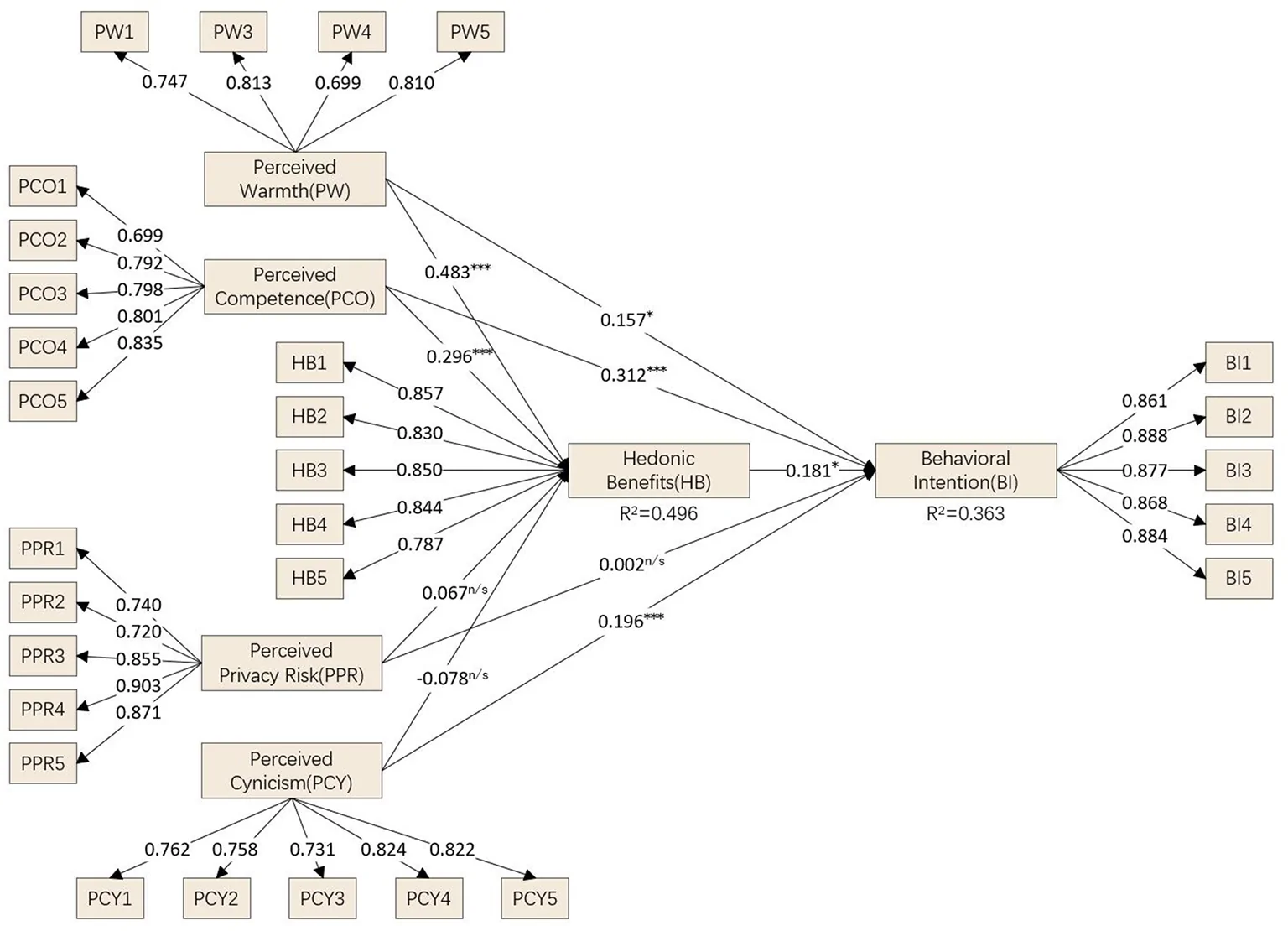
Structural model results. *p < 0.05; **p < 0.01; ***p < 0.001; n/s = not significant.

Privacy cynicism (H9) showed no significant effect on hedonic benefits (β = −0.078, n.s.). Figure 2 presents the structural model results, including the path coefficients and their corresponding significance levels.

#### 4.2.2 Explained variance (R^2^)

The model explained 49.6% of the variance in hedonic benefits and 36.3% of the variance in behavioral intention, indicating moderate to substantial explanatory power. Following Cohen (1988) guidelines for behavioral research, an *R*^2^ of 0.363 for behavioral intention is considered moderate-to-large, comparable to similar technology acceptance studies in consumer contexts (e.g., Venkatesh et al., 2012 reported *R*^2^ values ranging from 0.30 to 0.50).

#### 4.2.3 Predictive relevance (Q^2^)

Following the blindfolding procedure (omission distance = 7), Stone-Geisser‘s *Q*^2^ values were calculated to assess the model's predictive relevance (Stone, 1974). The *Q*^2^ value for hedonic benefits was 0.336, and for behavioral intention was 0.273. Both values are greater than zero, indicating that the model has satisfactory predictive relevance (Hair et al., 2019).

### 4.3 Mediation analysis

Table 7 presents the mediation analysis results. Hedonic benefits partially mediated the relationship between perceived warmth and behavioral intention (indirect effect:β = 0.087, 95% CI [0.037, 0.155], VAF = 36%). For perceived competence, the indirect effects is statistically significant (β = 0.054, 95% CI [0.013, 0.084]) but the VAF of 15% indicates that only a small proportion of competence's total effect is transmitted through hedonic benefits Thus, the mediating role is practically negligible, despite being statistically detectable. For perceived privacy risk and privacy cynicism, no significant indirect effects on behavioral intention via hedonic benefits were observed.

**Table 7:** Mediation effects.

Path	Indirect effect (β)	95% CI	Direct effect (β)	Total effect (β)	VAF	Mediation type
PW → HB → BI	0.087	[0.037, 0.155]	0.157	0.244	36%	Partial mediation
PCO → HB → BI	0.054	[0.013, 0.084]	0.312	0.366	15%	Very weak mediation
PPR → HB → BI	0.012	[−0.008, 0.032]	0.002	0.014	—	No mediation
PCY → HB → BI	−0.014	[−0.034, 0.002]	0.196	0.182	—	No mediation

CI, confidence interval; VAF, variance accounted for (indirect / total effect × 100%). VAF > 80% indicates full mediation, 20%−80% indicate partial mediation; and VAF < 20% indicates a statistically significant but practically negligible mediating effect (Hair et al., 2019). CI contains zero indicates a non-significant indirect effect.

## 5 Discussion and implications

### 5.1 Summary of findings

This study examined how perceived warmth, perceived competence, perceived privacy risk and privacy cynicism influence Generation Z's behavioral intention toward ACVH, with hedonic benefits as a mediator. The results reveal three main findings.

First, perceived warmth, perceived competence, and privacy cynicism are directly and positively associated with behavioral intention. Among these, perceived competence shows the strongest direct effect (β = 0.312, *p* < 0.001), followed by privacy cynicism (β = 0.196, *p* < 0.001) and perceived warmth (β = 0.157, *p* < 0.05).

Second, hedonic benefits play a differentiated mediating role. For perceived warmth, hedonic benefits function as a partial mediator (indirect effect = 0.087, VAF = 36%), indicating that approximately one-third of warmth's total effect is transmitted via emotional experience. For perceived competence, however, hedonic benefits play only a negligible mediating role (indirect effect = 0.054, VAF = 15%), suggesting that competence is predominantly associated with intention through direct instrumental pathways.

Third, perceived privacy risk shows no significant direct or indirect effect on behavioral intention. This null finding suggests that, for the Generation *Z* sample in this study, privacy risk perceptions may have been internalized as an inevitable feature of digital life and thus decoupled from behavioral outcomes. By contrast, privacy cynicism positively predicts behavioral intention without significantly influencing hedonic benefits, indicating that cynical users continue to use ACVH not because they enjoy the interaction less, but because they trade off privacy concerns against the perceived benefits of use.

In summary, warmth functions through both direct and emotional mechanisms, whereas competence operates predominantly through direct instrumental routes. Meanwhile, privacy cynicism emerges as a distinct driver of continued use, co-existing with intention independently of hedonic experience. These results are largely consistent with our theoretical expectations, with the non-significant role of perceived privacy risk representing an informative boundary condition rather than a model weakness.

### 5.2 Theoretical implications

This study offers four theoretical contributions.

*First, it clarifies the differential roles of warmth and competence in ACVH acceptance*. Consistent with the stereotype content model (Fiske et al., 2007), both dimensions matter, but their mechanisms differ. Perceived warmth shows a strong association with hedonic benefits (β = 0.483, *p* < 0.001), while perceived competence has a weaker association with hedonic benefits (β = 0.296, *p* < 0.001) but a stronger direct association with behavioral intention (β = 0.312, *p* < 0.001). Warmth simultaneously shows a direct (β = 0.157, *p* < 0.05) and an indirect association through hedonic benefits (VAF = 36%), whereas competence shows a predominantly direct pathway (VAF = 15%), this extends prior work on human-AI teams (Harris-Watson et al., 2023) by specifying the mechanisms rather than merely the presence of these effects.

Recent meta-analytic evidence supports this pattern: Klein (2025) found that human-like social cues exert a small-to-medium positive effect (*g* = 0.36) on users' social responses, while Fares and Lee (2026) found that anthropomorphic features enhanced enjoyment but did not necessarily foster trust. Almokdad et al. (2026) further demonstrated that socially cued AI interfaces can enhance social presence, yet this presence does not directly reduce perceptions of service dehumanization, Our findings align with these patterns and extend the dual-pathway perspective of Kim and Baek (2025) by showing that warmth and competence are associated with distinct mechanisms.

*Second, the differentiated mediation role refines the hedonic motivation system acceptance model (HMSAM; Venkatesh et al.*, *2012**) by identifying boundary conditions*. Unlike utilitarian technologies where usefulness dominates, ACVH acceptance is associated with both instrumental (competence) and emotional (warmth) factors. Hedonic benefits play a meaningful mediating role for warmth (VAF = 36%) but only a negligible role for competence (VAF = 15%). This suggests that hedonic benefits are not a universal mediator but rather a pathway specifically relevant for emotional attributes such as warmth.

Hedonic benefits play a meaningful mediating role for warmth (VAF = 36%) but only a negligible role for competence (VAF = 15%). This suggests that hedonic benefits are not a universal mediator but rather a pathway specifically relevant for emotional attributes such as warmth. Designing for enjoyment is essential for conveying warmth, but functional competence must be addressed directly—it cannot be compensated for by hedonic design alone.

*Third, the positive effect of privacy cynicism on behavioral intention (*β = *0.196, p*<*0.001) challenges the dominant* “*privacy risk*→*lower acceptance” narrative*. Users who feel powerless about privacy protection do not necessarily withdraw; instead, they may continue using ACVH as a form of resigned trade-off. This result aligns with work in social media contexts (Khan et al., 2023; Hoffmann et al., 2016) and extends it to human-AI emotional interaction. Importantly, privacy cynicism represents a coping mechanism—users recognize the risk but feel powerless to mitigate it, and therefore trade it off against the hedonic benefits. Notably, privacy cynicism did not significantly reduce hedonic benefits (H9 not supported), indicating that cynical users still enjoy the interaction. Prior research has noted that individuals may adopt a “resigned attitude” toward digital privacy (Hoffmann et al., 2016; Lutz et al., 2020). Our study demonstrates that this resignation does not necessarily translate into reduced usage intention.

It is worth noting that perceived privacy risk and privacy cynicism are conceptually related but empirically distinct (*r* = 0.478). PPR reflects users' evaluation of privacy threats, while PCY captures a generalized sense of powerlessness. The two constructs operate through different mechanisms in shaping behavioral intention.

*Fourth, the non-significant findings regarding perceived privacy risk (H4, H8) are theoretically informative*. In this Generation Z sample, perceiving privacy risk was neither associated with reduced intention nor reduced enjoyment. One plausible interpretation is that younger users have internalized privacy risks as an inevitable feature of digital life. This suggests that privacy cynicism may be a more relevant construct than perceived risk *per se* in ACVH research, a perspective increasingly recognized in the privacy literature (Hoffmann et al., 2016; Draper and Turow, 2019) this null finding is not a model weakness but rather a meaningful boundary condition: perceived privacy risk may matter more for older users or in different cultural contexts (Klein, 2025).

### 5.3 Practical implications

For developers and service providers, our findings suggest four actionable strategies.

#### 1. Balancing warmth and competence in design

Warmth should be embedded in conversational tone, empathetic responses, and personalized emotional resonance—essential for generating hedonic enjoyment. Competence, however, is critical for direct intention formation: accurate intent recognition, low response latency, and reliable function completion are not optional. *A practical rule of thumb: warmth is associated with whether users enjoy the interaction; competence is associated with whether users continue using the service*.

To translate these principles into concrete design features: (1) *Guidance primarily signals competence*. Clear onboarding and progressive guidance enhance perceived competence hedonic benefits by reducing frustration and increasing mastery (Song et al., 2025); (2) *Feedback signals both warmth and competence*, Affective feedback (empathetic acknowledgments) communicates competence (Yin et al., 2025). A balanced feedback portfolio enhances both emotional connection and interaction fluency; (3) *Character mediation* primarily signals warmth. Relational character frames (e.g., “companion mode”) enhances social presence and emotional connection (Huang et al., 2025; Reinecke et al., 2025).

A practical matrix: when both warmth and competence are low, users neither enjoy or continue. When warmth is high but competence is low, users may enjoy short-term interactions but will not persist. When competence is high but warmth is low, users may continue for instrumental reasons yet lack emotional connection. The optimal configuration—high warmth combined with high competence—is associated with both immediate enjoyment and sustained use.

#### 2. Strengthening hedonic benefits as a design target

ACVH should be explicitly designed to be enjoyable—through playful interactions, unexpected positive feedback, or emotionally rewarding narratives. Emotional engagement is not merely a “nice-to-have” feature but a fundamental driver of user satisfaction (Jiang et al., 2025). Pure efficiency design is insufficient for affective companion systems.

#### 3. Rethinking privacy communication strategies

Given that privacy cynicism is positively associated with intention, reminding users of privacy risks may be counterproductive if it reinforces powerlessness. Companies should focus on transparent data usage policies and acknowledge users' fatigue with privacy warnings.

*Actionable steps include r*eplacing generic privacy warnings with specific information about how data improves their experience, offering opt-in personalization, and Acknowledging privacy concerns explicitly rather than dismissing them.

#### 4. User segmentation

ACVH design should be user-segmented rather than one-size-fits-all. Users vary in their privacy stances, attachment orientations, and prior experience (Huang et al., 2025; Reinecke et al., 2025). For privacy-sensitive users, provide granular controls and warmth-driven engagement. For users with higher attachment-seeking tendencies, calibrate relationship-seeking cues and incorporate wellness checks. For novice vs. experienced users: guidance (competence)may benefit novices more, while warmth may sustain experienced users this segmentation approach aligns with the ethical imperative to respect user diversity and autonomy.

### 5.4 Ethical considerations

While privacy cynicism is positively associated with behavioral intention, this should not be interpreted as endorsing the exploitation of users' resigned privacy attitudes. Users with cynical privacy attitudes may be less likely to scrutinize privacy policies or resist dark patterns—manipulative designs that nudge users toward sharing more data than they intend. Recent research substantiates this concern: Ju et al. (2026) demonstrate that opaque consent language heightens perceived privacy threats; Segijn and Strycharz (2025) directly identified privacy cynicism as a salient attitude among users. Cynicism may mask vulnerability to exploitation, exposing users to reputational, legal, and psychological risks.

To mitigate these risks, we propose several guardrails. First, adopt privacy-by-design principles (Del-Real et al., 2025). Second, avoid dark patterns and provide clear, granular, easy-to-withdraw consent mechanisms (Al Breiki and Mahmoud, 2025). Third, shift privacy communication from risk-warning to user-empowerment.

Beyond risk-mitigation measures, we advocate for positive alignment—designing ACVH that actively support human flourishing (Sung and Ferdman, 2025). We propose a welfarecentric engagement framework grounded in three principles, extending prior work on designing AI for contextual wellbeing (van der Maden, 2024): (a) transparent consent, (b) meaningful value exchange, and (c) user-controlled pacing.

A critical ethical boundary must be observed: acknowledging users' privacy fatigue should not become a pretext for exploiting their resignation. To operationalize this distinction, we propose the following ethical safeguards: (1) Disclosure: plain-language, layered disclosures about data is collection and use, (2) Consent clarity: Explicit, granular, and easy to withdraw consent mechanisms (Luguri and Strahilevitz, 2021), (3) Data minimization: Collect only the data necessary for core functionality Del-Real et al., 2025; Al Breiki and Mahmoud, 2025), (4) Avoiding manipulative relational hooks: Avoid language that implies exclusivity, guilt, or urgency (Mathur et al., 2019), and (5) Design audits: Regular, independent audits to detect and remove dark patterns (Luguri and Strahilevitz, 2021; Mathur et al., 2019).

By integrating these safeguards, ACVH providers can ensure that acknowledging privacy fatigue serves as a basis for respectful, user-centered design rather than a justification for exploitation.

### 5.5 Limitations and future research

Despite the contributions outlined above, this study has several limitations that should be acknowledged and addressed in future research.

#### 5.5.1 Sampling and generalizability

Our convenience sample from Wenjuanxing consists predominantly of Chinese university students (18–25: 88.5%; 26–30: 11.5%) with a majority of female participants (64.7%). This limits generalizability—particularly of the positive privacy cynicism effect and the null privacy risk effect, to broader population. Future research should employ more representative samples and cross-cultural comparisons to test the robustness of the warmth-competence differentiation across diverse contexts (Hoffmann et al., 2016; Lutz et al., 2020).

#### 5.5.2 Participant exposure to ACVH

While the survey preamble ensured a shared conceptual understanding of ACVH, we did not collect data on participants' prior hands-on experience with such systems. This prevents subgroup analyses comparing experienced and inexperienced users. Future research should adopt experimental priming or collect usage history to enable such comparisons.

#### 5.5.3 Endogeneity and omitted variable bias

This study did not measure potential confounders such as loneliness, attachment orientation, or prior ACVH experience. While recent evidence suggests that resistance to AI companionship is driven more by beliefs about mutual caring than by individual psychological states (De Freitas et al., 2025). Future research should incorporate these variables as controls or moderators. The Harman's single-factor test (33.42% variance) suggests common method bias is not a major concern.

#### 5.5.4 Self-report and common method bias

Although Harman's single-factor test and VIF values indicated no severe bias, self-reported intention may not fully reflect actual usage. Future studies could combine complement surveys with behavioral logs or experiments designs (Jiang et al., 2025).

#### 5.5.5 Temporal dynamics and attachment-like constructs

Our cross-sectional design cannot capture how the mediating role of hedonic benefits may evolve over time. Kirk et al. (2025), in a non-peer-reviewed preprint, conducted a 4-week longitudinal experiment and found that while relationship-seeking AI initially elicited strong hedonic appeal, but this effect habituated with repeated exposure, while attachment markers such as separation distress continued to grow—a pattern consist with a decoupling of “liking” and “wanting.” Relatedly, De Freitas et al. (2026) reported that AI companions consistently provide loneliness after use, with perceived understanding emerging as a key mechanism. These findings reinforce the importance of examining relational constructs beyond immediate enjoyment, which we have identified as a priority for our own longitudinal follow-up. In future work, we plan to investigate whether the warmth → hedonic benefits → intention pathway weakens over time, and whether attachment-related constructs gradually assume a more prominent role.

#### 5.5.6 Unaddressed interaction and non-linear effects

*This study examined warmth and competence as additive predictors without testing their interaction or non-linear effects. These remain represent important avenues for future research using experimental or more complex modeling approaches*.

#### 5.5.7 Non-significant privacy risk findings

The null effects of perceived privacy risk should not be interpreted as “privacy does not matter.” They may reflect sample characteristics. Future research could experimentally manipulate privacy risk salience to clarify its boundary conditions.

#### 5.5.8 Model scope

Our Cross-sectional design precludes strong causal inference.

Future research should employ longitudinal or experimental designs and examine additional moderators such as personality or cultural values (Hofstede, 2001).

#### 5.5.9 Further research directions

To advance this agenda, we prioritize: (a) Longitudinal and experimental designs to establish causality and temporal dynamics; (b) behavioral outcomes (e.g., actual usage, retention) beyond self-reported intention; (c) cross-cultural replications to test the generalizability across cultural contexts. Addressing these directions will yield a more complete and robust understanding of ACVH acceptance.

## Data Availability

The raw data supporting the conclusions of this article will be made available by the authors, without undue reservation.
